# Somnoforme: A New Anæsthetic

**Published:** 1902-10

**Authors:** 


### Reviews of Dental Literature.

Somnoforme: A New Anæsthetic.—At the last meeting of the British Dental Association, at Shrewsbury, May, 1902, a paper was read with the above title, prepared by Drs. Rolland and Robinson, of Bordeaux.

Somnoforme is said to be composed of sixty parts chloride of ethyl, thirty parts chloride of methyl, and five parts bromide of ethyl. While some little care is needed in its preparation, the formula is not secret. It is credited to Dr. Rolland, Professor of Anæsthesia at the Bordeaux Dental School. It is claimed to be safe, readily used, quickly producing its effect, followed by quick recovery without unpleasant after trouble, and especially useful in dental and other like short operations. Its effects can be continued as long as needed, and it may be repeatedly administered without risk, if on recovery the operation is found to be incomplete.

The apparatus for its administration is very simple, an ordinary handkerchief folded so as to form a cone-shaped mask, in the folds of which is placed a piece of paper to prevent evaporation, the apex of the cone being completely filled with a piece of cotton-wool. Upon the cotton is sprayed from five to ten cubic centimetres of somnoforme, five being sufficient for ordinary cases; the cone thus charged with somnoforme is placed over the face, covering the mouth and nose completely, and as far as possible no air being allowed to pass. No precautions are needed as to abstaining from food before its administration; or regarding position; nor is it necessary to loosen the collar or waistband, or, in the case of ladies, to undo stays or loosen their underclothing.

The advantages claimed are, no cumbersome apparatus; instantaneous action; rapid elimination and rapid return of consciousness and use of faculties; security both in the beginning, during, and after effects.

In answer to questions during the discussion of the paper, Dr. Robinson said the stability of the drug could be maintained provided the stopper was hermetically sealed. Dr. Rolland suggested that it might be best to have it put up in glass capsules containing just enough for a dose. It is necessary to exclude air during its administration, to avoid trouble from excitement of the patient. With regard to its danger limits, he said, "Any anæsthetic is bound to kill if continued long enough." He had found a cat in the street which had had its back broken by a dog; he kept the cat under the effects of somnoforme for eight consecutive hours; during the entire time it was in an anæsthetic sleep; it was then killed by an overdose. (*Dental Record,* London, July, 1902, page 312.)

In Ash & Sons' Quarterly Circular, June, 1902, page 148, we have a report from Dr. Pinet, Professor of Anæsthesia at the Dental School of Paris, and Ch. Jeay, chief of the clinic at the same school, giving their experience in the use of this new anæsthetic. The formula is slightly different,—chloride of ethyl, 60; chloride of methyl, 35; bromide of ethyl, 5. In the experiments related, anæsthesia was produced in from thirty to forty seconds, as a rule, and lasted about thirty to ninety seconds. In some cases it was all that could be desired, in others there was some excitement, which in a few cases was quite violent; unpleasant after effects were in some cases noted.

The excitement in some cases seemed to be due to a want of air, a true "air hunger;" and in others it is ascribed to a leakage of air under the mask or cone, which retards the anæsthetic effect. To remedy this Dr. de Crésantignes has invented an apparatus which he calls a "face-piece holder with a bladder." "This extremely ingenious apparatus allows the patient half a litre of non-renewable physiological air, which is indispensable for satisfying the imperious demand of the patient to fill his lungs."

"Thanks to this apparatus, the patient respires freely, does not struggle, and the close-fitting recommended by Dr. Rolland is obtained much more easily than with the old face-piece."

This small amount of air assists the rapid action of the anæsthetic without unduly diluting it, and it is carried into the lungs with the air; while with a close-fitting mask, without this provision, the patient, being unable to breathe freely, fails to take in the requisite amount of somnoforme vapor to quickly produce the desired effect.

It will probably be some time before the practical value of this new anæsthetic in its relation to dental practice can be determined. In the mean time it is well to observe caution until in skilful hands its merits and demerits are scientifically investigated, and its advantages, if any, over other accepted safe anæsthetics have been demonstrated by actual use. It is to be borne in mind that, of all anæsthetics, the chlorine combinations are most dangerous.

